# Schizophrenia patient-derived olfactory neurosphere-derived cells do not respond to extracellular reelin

**DOI:** 10.1038/npjschz.2016.27

**Published:** 2016-08-17

**Authors:** Jing Yang Tee, Ratneswary Sutharsan, Yongjun Fan, Alan Mackay-Sim

**Affiliations:** 1 Eskitis Institute for Drug Discovery, Griffith University, Brisbane, QLD, Australia

## Abstract

Reelin expression is reduced in various regions in the post-mortem brain of schizophrenia patients but the exact role of reelin function in the neurobiology of schizophrenia remains elusive. Absence of reelin in knockout mouse causes inverted lamination of the neocortex due to aberrant neuronal migration. The aim of this study was to utilize patient-derived olfactory neurosphere-derived (ONS) cells to investigate whether extracellular reelin alters cell motility in schizophrenia patient-derived cells. ONS cells from nine patients were compared with cells from nine matched healthy controls. Automated high-throughput imaging and analysis were used to track motility of individual living cells on reelin-coated surfaces produced from reelin secreted into the medium by HEK293FT cells transfected with the full-length reelin plasmid pCrl. Automated assays were used to quantify intracellular cytoskeleton composition, cell morphology, and focal adhesions. Expression of reelin and components of the reelin signaling pathway were measured by western blot and flow cytometry. Reelin inhibited the motility of control cells but not patient cells, and increased the number and size of focal adhesions in control cells but not patient cells. Patient and control cells expressed similar levels of the reelin receptors and the reelin signaling protein, Dab1, but patient cells expressed less reelin. Patient cells were smaller than control cells and had less actin and acetylated α-tubulin, components of the cytoskeleton. These findings are the first direct evidence that cellular responses to reelin are impaired in schizophrenia and are consistent with the role of reelin in cytoarchitectural deficits observed in schizophrenia patient brains.

## Introduction

Reelin gene (*RELN*) encodes a large secreted glycoprotein whose signaling pathway is implicated in neurotransmission,^1^ synaptic plasticity,^2,3^ and memory formation.^2,4^ Reelin protein and mRNA expression are reduced in various regions of the post-mortem brain in schizophrenia patients, e.g., prefrontal cortex, temporal cortex, cerebellum, hippocampus, and caudate nucleus.^5–9^ The concurrent observation of reduced reelin expression and abnormal interstitial neuron distribution in the same superficial white matter region in schizophrenia patient brains led to speculation that reelin could have a key role in brain development and thus schizophrenia disease etiology.^5^ A “reelin hypothesis” is proposed in the etiology of schizophrenia in which the reduced levels of reelin would affect the trajectory of brain development leading to schizophrenia-associated differences in brain structure and function.^9^ Reduced reelin in the adult brain may also be consequential because reelin is continuously produced in the olfactory bulb^10^ and the dentate gyrus of the hippocampus.^11^

Extracellular reelin signals via a dedicated intracellular signaling pathway. Secreted reelin binds two cell membrane receptors, apolipoprotein E receptor 2 (*ApoER2*), and very low-density lipoprotein receptor (*VLDLR*)^12^ to activate accessory protein disabled-1 (*Dab1*) via phosphorylation,^13^ which then triggers the reelin signaling cascade. Reelin may also activate focal adhesion kinase (*FAK*) pathway via interaction with integrin receptors α3β1 (ref. 14) and α5β1.^15^ Focal adhesions are cell membrane structures that link the extracellular matrix (ECM) with the intracellular actin cytoskeleton and are essential for cell adhesion and motility.^16^ The FAK signaling pathway, which regulates cell adhesion to extracellular proteins via integrin receptors, is impaired in schizophrenia patient-derived olfactory cells.^17^ The exact mechanism of reelin-dependent modulation of cell migration remains debatable. Some studies have suggested that reelin triggers a “detachment signal”^18^ or a “stop signal”^14,19^ to inhibit radial migration, postulated to be a by-product of increased filamentous actin stabilization in the presence of secreted reelin.^20^ Conversely, reelin acts as a chemoattractant that stimulates newborn neurons to move towards the cortical surface in the neocortex,^21^ more recently modeled *in silico*,^22^ and supported by evidence that reelin activates growth cones and filopodia,^23^ structures that depend on active modulation of the actin and microtubule cytoskeleton.

The aim of the present study was to investigate whether extracellular reelin alters cell motility in schizophrenia patient-derived olfactory cells.^17,24,25^ Gene expression profiling of patient olfactory cells indicated that expression of many genes in the reelin signaling pathway were significantly affected in schizophrenia, including reduced *RELN* expression, compared with healthy control cells.^24^ These cells may have less intracellular reelin and be impaired in their response to extracellular reelin. Cell motility was quantified in the presence of extracellular reelin using automated imaging and analysis of living cells in a 96-well format, providing a non-biased quantification of large numbers of cells from nine patients with schizophrenia and nine healthy controls. Automated image analysis was also used to quantify the number and size of focal adhesions and expression of cytoskeletal proteins, actin, and acetylated α-tubulin. The results demonstrate the first direct evidence for the effects of extracellular reelin in cell migration in schizophrenia.

## Results

### Patient cells have less endogenous reelin

**Figure 1:**
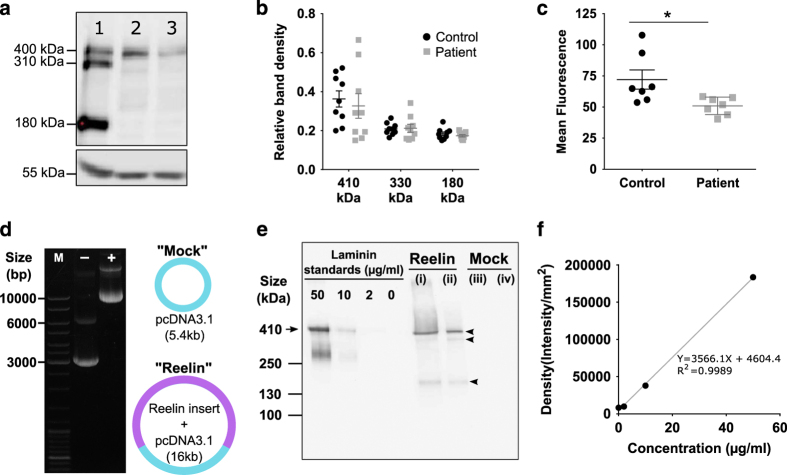
Patient-derived cells have reduced endogenous reelin and recombinant full-length reelin production into conditioned medium. (**a**) Qualitative representation of reelin expression by western blot showing total protein samples obtained from (1) mouse brain lysate, (2) healthy control olfactory neurosphere-derived (ONS) cell line and (3) schizophrenia patient ONS cell line. Mouse brain lysate was used as a positive control and β-tubulin (55 kDa) was the loading control. (**b**) Quantitation of reelin expression by western blot. Reelin band densities were divided with β-tubulin band densities to obtain relative band intensities. Relative band intensities were presented as mean±s.e.m. (**c**) Flow cytometry quantitation of reelin expression in fixed ONS cells. Fluorescence intensities for 10,000 cells stained with anti-reelin antibody followed by Alexa Fluor 488 were measured via flow cytometer and normalized to isotype-matched IgG antibody. (**d**) Amplification and purification of full-length reelin plasmid pCrl and mock vector pcDNA3.1. Plasmids were verified by ethidium bromide agarose gel. GeneRuler DNA ladder mix was used as the size marker (“M”); other two lanes contained “Mock” vector pcDNA3.1 (−) and “Reelin” plasmid pCrl (+). (**e**) Verification of recombinant full-length reelin. Conditioned medium was collected from HEK293FT cells transfected with either the “Reelin” pCrl plasmid or “Mock” plasmid. Recombinant mouse laminin (molecular size 410 kDa; arrow) was used as a standard to validate full-length reelin size and to estimate concentrations present. Conditioned medium was purified and concentrated with Amicon-15 centrifugal tubes. Unpurified samples presented in lanes (i) and (iii) for reelin and mock-conditioned medium, respectively. Purified reelin and mock-conditioned medium were in lanes (ii) and (iv). Reelin bands were probed with anti-reelin antibody to give the full-length band (410 kDa) and its isoforms, highlighted with arrowheads. (**f**) Laminin standard curve to correlate western blot band density (*y *axis) with protein concentration (*x* axis). All data were presented as mean per group±s.e.m. Each point in the scatter plot represents data for each cell line used. *Student’s *t*-test**,* P*=0.028.

By denaturing total cell protein samples and running samples on a reducing polyacrylamide gel, we identified the key reelin fragments that are widely regarded as the full-length reelin (~410 kDa) and the 310 and 180 kDa isoforms (Figure 1a) via western blot. Reelin expression was a normalized value between reelin band densities divided by β-tubulin band densities (Figure 1b). Patient cells had marginally less full-length reelin protein (0.327±0.063) compared with control cells (0.363±0.042), however, this difference was not statistically significant due to the marginal overlap between individual samples. Patient cells also had similar levels of reelin 310 and 180 kDa isoforms. It is noteworthy that western blot is semi-quantitative and lacks the sensitivity to detect subtle changes in expression. Next, we used flow cytometry to verify our western blot observations, by quantifying reelin immunofluorescence of single cells in suspension. Cells were fixed and probed with a highly specific antibody against full-length reelin. We made the assumption that secondary fluorophore-conjugated antibody staining levels, measured as mean fluorescence index (MFI), were direct representation of reelin expression. In agreement with the western blot results, patient cells have less reelin content (MFI 50.95±2.65), which significantly differed to healthy control levels (MFI 72.07±7.72); *P*=0.024, *t*=2.59, df=12 (Figure 1c). These findings support the reduced reelin expression reported for post-mortem patient brains, hence, supports the use of our proposed olfactory neurosphere-derived (ONS) cell model to probe further into the cellular mechanisms of the effects of reelin in schizophrenia.

### Production of full-length reelin into conditioned medium

In order to investigate the effects of reelin on patient-derived cells, we had to first produce recombinant full-length reelin, which is unavailable commercially, by transfection of the well-published pCrl reelin-containing plasmid (Figure 1d). pCrl contains the full-length reelin gene sequence (~11 kb) in a pcDNA3.1 vector (5.4 kb) (Figure 1d). Both “Mock” vector pcDNA3.1 (−) and “Reelin” plasmid pCrl (+) show correct sized bands that includes both supercoiled and circular plasmid forms. Supercoiled plasmids were relatively smaller in size compared with circular uncoiled forms: 3 kb for pcDNA3.1 (−) and 10 kb for pCrl (+) (Figure 1d). Reelin was successfully produced, purified, and concentrated from conditioned medium secreted by immortalized HEK293FT cells (Figure 1e). Concentration of full-length reelin in the final stock was estimated via standard curve of increasing laminin concentrations (0, 2, 10, 50 μg/ml laminin; Figure 1f). By using resulting standard curve formulae, full-length reelin band density (lane (ii) Figure 1e) corresponded to estimated protein concentrations of 38.25 μg/ml or 0.612 μg. Reelin was not present in mock-conditioned medium from HEK293FT cells transfected with pcDNA3.1 plasmid without reelin insert (lanes (iii) and (iv) in Figure 1e), to show that it is an appropriate assay control.

### Patient cells were unable to modulate cell motility in response to extracellular reelin

**Figure 2:**
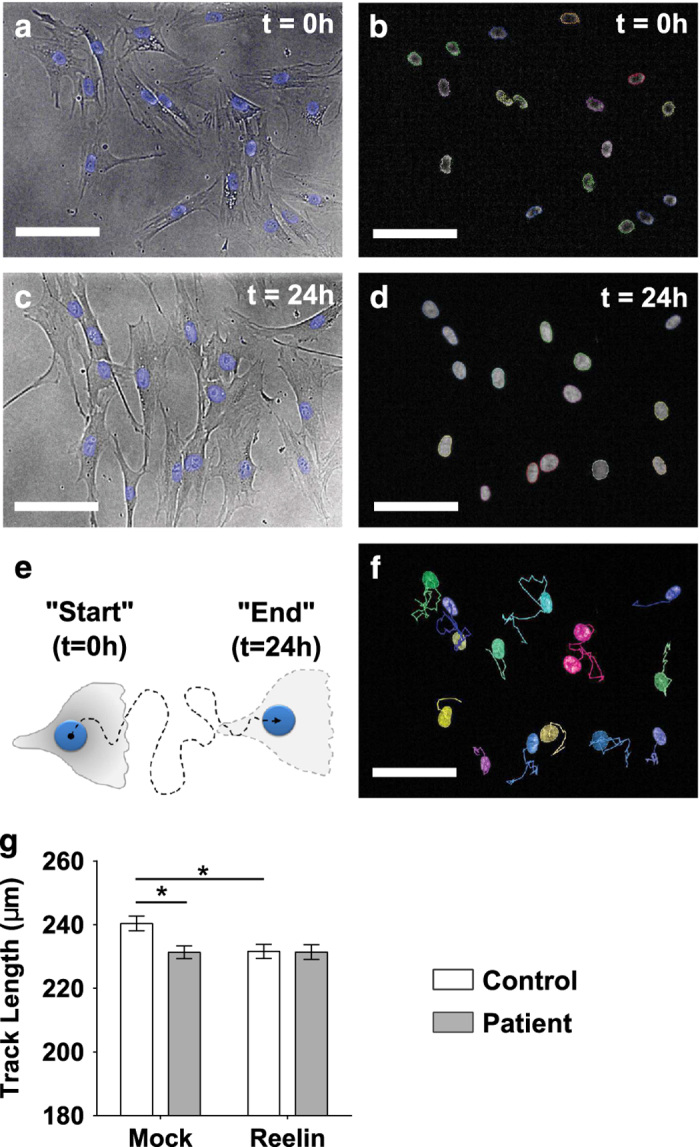
Patient-derived cells did not respond to extracellular reelin to alter cell motility. Live cells were stained with NucBlue dye to visualize cell nuclei, which was tracked over 24 h to measure cell motility. Age-matched patient cell lines (*N*=9) were compared with control cell lines (*N*=9). For each cell line, at least 25 single cells were tracked throughout the whole 24 h duration to generate 25 unique tracks. (**a**) Representative brightfield image overlaid with NucBlue stained nuclei imaged straight after incubation with NucBlue dye; *t*=0 h. (**b**) Harmony software (PerkinElmer, Llantrisant, UK) identified all cell nuclei stained with NucBlue dye based on “Find nuclei” search algorithm; *t*=0 h. (**c**) Live cells retain the NucBlue stain after 24 h, imaged as the final frame at the end of the single cell migration assay. (**d**) Representative image of “Find nuclei” algorithm used to identify all NucBlue stained nuclei at *t*=24 h. (**e**) Single cells were tracked for a total duration of 24 h by imaging multiple field of views at one frame per 30 min. Cell motility was quantified by measuring frame-to-frame movement of cell nuclei from “Start” (first imaged frame; *t*=0 h) to “End” (last imaged frame; *t*=24 h). (**f**) Harmony software was used to generate motility tracks of each single cell to represent the accumulated distance covered by each cell over 24 h. (**g**) Effects of extracellular reelin on olfactory neurosphere-derived cells. Wells were coated with purified reelin or mock-conditioned medium. All assays were run in the same instance in a 96-well plate format. Motility of patient cells (closed gray bars) were directly compared with age-matched healthy control cells (open bars), in the absence (“Mock”) or presence (“Reelin”) of reelin. All plotted data represents mean of all cells measured per group±s.e.m. *P<0.05 based on Tukey’s *post-hoc* test following two-way ANOVA. Scale bar = 100 μm.

To elucidate the role of reelin in patient cell migration, a single cell tracking assay was used to compare motility of patient cells compared with control cells on reelin-coated and reelin absent well surfaces. Cells were stained with NucBlue to probe nuclei in living cells (Figure 2a,c), which allows accurate tracking of cells by using the nuclei as reference points. Cell motility was measured as the accumulated distance traveled by an individual cell imaged every 30 min over a total duration of 24 h, from 0 h after seeding (Figure 2b) to 24 h upon assay completion (Figure 2d), which generates unique motility tracks for each individual cell (Figure 2f).

For each cell line, at least 25 single cells were tracked throughout the whole 24 h duration to generate 25 unique tracks. All measured tracks were cumulatively averaged to give the mean track length for the disease group versus healthy control group (results presented in Figure 2g). Two-way ANOVA used to estimate the main effects of reelin supplementation, disease association and interaction between reelin×disease status, showed a significant impact on cell motility. Resulting test results indicated both reelin coat (*P*=0.048, F(1, 2261)=3.92) and disease status (*P*=0.048, F(1, 2261)=4.41) significantly influenced cell motility. The interaction between disease status and reelin was also a confounding factor that modulated cell motility (*P*=0.046, F(1, 2261)=4.01). *Post-hoc* Tukey’s multiple comparison tests were conducted to estimate if mean track lengths were significantly different between groups.

A closer examination of data revealed that patient cells moved shorter distances (231.34±1.99 μm) compared with control cells (240.37±2.30 μm) in the absence of reelin, where distance traveled by patients significantly differed to control tracks (*P*<0.05). On reelin-coated surfaces, control cells responded by reducing track lengths by ~10μm to 231.61±2.20 μm (*P*<0.05). In contrast, patient cells did not show any responsive changes to motility in the presence of reelin by moving similar distances as that observed in mock surfaces (*P*>0.05). Besides the apparent non-responsive phenotype, it is noteworthy that reelin stimulus brought cell motility in control cells down to patient levels. Because we are dealing with micrometer level differences, changes in track length was only detectable by software-assisted analysis, and not observable by eye balling resulting track images. Hundreds of tracks from multiple cell lines in different coating conditions were screened by software to eliminate human bias.

### Patient cells express reelin receptors and key reelin signaling adaptor protein

**Figure 3:**
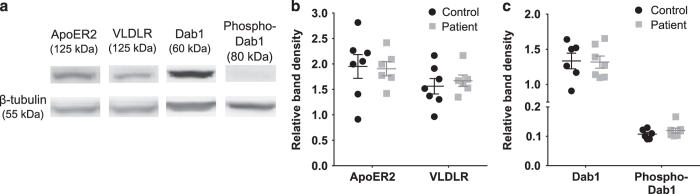
Patient-derived cells expressed key components of the reelin pathway. (**a**) Representative western blot staining for reelin receptors apolipoprotein E receptor 2 (ApoER2), very low-density lipoprotein receptor (VLDLR), accessory protein Dab1, and phosphorylated Dab1 (phospho-Dab1). (**b**) Quantitation of reelin-binding receptors—ApoER2 and VLDLR in whole-cell lysates, normalized to β-tubulin. (**c**) Quantitation of Dab1 and phosphorylated Dab1 in whole-cell lysates, normalized to β-tubulin. For all graphs, control dataset was presented as black circles and patient dataset were gray squares. All data were presented as mean per group±s.e.m. Each point in the scatter plot represents data for each cell line used.

Next, we were interested to know if ONS cells will be able to respond to reelin via receptor binding and initiation of reelin signaling cascade. Western blot was used to quantify expression of the two well-published reelin receptors—ApoER2 and VLDLR, both with expected band fragments at molecular size ~125 kDa (Figure 3a). Both patient and control cells express equal amounts of ApoER2 and VLDLR. Reelin receptor binding leads to the recruitment of the crucial adaptor protein Dab1 to the transmembrane region of both reelin receptors. Dab1 protein was present in both patient and control cell lines, with a specific band at the correct molecular weight (~60 kDa; Figure 3a). We also showed that both patient and control cells were able to activate Dab1 equally via phosphorylation of Dab1 protein at tyrosine 198 (band detected at ~80 kDa). All band densities were normalized with corresponding β-tubulin band densities (~55 kDa; Figure 3a) to estimate protein levels. Expression levels of ApoER2 and VLDLR in patient cells did not significantly deviate from levels measured in healthy control cells (student’s *t*-test, *P*>0.05; Figure 3b). Patient cells expressed same levels of Dab1 protein as control cells, and was able to phosphorylate Dab1 to activate the reelin signaling pathway (student’s *t*-test, *P*>0.05; Figure 3c). These findings suggest that patient cells are predicted to have the same capacity to bind to extracellular reelin via ApoER2 and VLDLR, followed by initiation of reelin pathway via Dab1.

### Reelin does not affect cell morphology and cytoskeletal content

**Figure 4:**
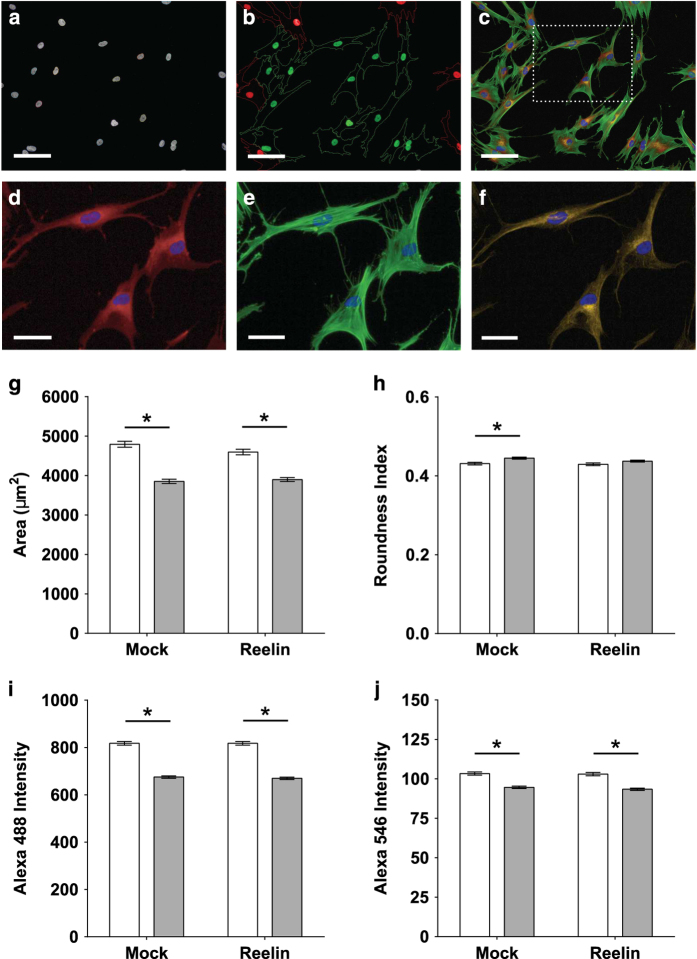
Reelin does not affect cell morphology and cytoskeletal content. Patient and control cells were fixed in 4% paraformaldehyde (PFA) stained for cell nuclei with 4′,6-diamidino-2-phenylindole (DAPI) and cytoplasm with CellMask Deep Red stain. Specific probes were used to visualize cytoskeletal proteins in actin and microtubules. Images were captured on Operetta High Content Imaging System (PerkinElmer, Llantrisant, UK) and analyzed by Harmony software. Age-matched patient cell lines (*N*=9) were compared with control cell lines (*N*=9). For all individual cell lines, 120 single cells were quantified and grouped into subsequent disease or non-diseased dataset. (**a**) “Find nuclei” algorithm was used to locate all cell nuclei stained with DAPI to identify all cells within the imaged field of views. (**b**) “Select population” algorithm was used to filter for cells that are fully within the field of view (green highlight) and omit cells partially in the field (red highlight). (**c**) Representative image of cells within a random field of view co-stained with DAPI (blue; nuclei), CellMask (red; cell cytoplasm), Alexa Fluor 488-conjugated phalloidin (green; filamentous actin), and anti-acetylated α-tubulin antibody followed by Alexa Fluor 546 secondary antibody (yellow; stable microtubules). (**d**) CellMask staining allows measurement of area covered by cell cytoplasm and cell roundness. (**e**) Filamentous actin expression levels were quantified based on mean fluorescence intensity of Alexa Fluor 488 conjugated to phalloidin within cell cytoplasm stained with CellMask. (**f**) Microtubule stability was measured based on expression levels of acetylated α-tubulin within cell cytoplasm stained with CellMask. (**g**) Quantitation of cell area, which correlates to the size of the cell. (**h**) Cell roundness was quantified as a relative roundness index where an index of “1” represents a perfect circle and “0” represents a straight line. (**i**) Actin expression graphs measured as Alexa Fluor 488 fluorescence intensities. (**j**) Stable microtubule expression measured as Alexa Fluor 546 fluorescence intensities. All plotted data represents mean of all cells measured per group±s.e.m. Open bars represent control cell dataset; closed gray bars represent patient cell dataset. Scale bar=50 μm. *P<0.05 based on Tukey’s *post-hoc* test following two-way ANOVA.

Next, we examined if non-responsive motility correlated with defects in cell morphology and cytoskeleton. Automated microscopy was used to capture images of fixed cells that were stained with fluorescent probes for cell cytoplasm, filamentous actin, and acetylated α-tubulin. For all individual cell lines, 120 cells were quantified to generate data presented in Figure 4g–j. 4′,6-Diamidino-2-phenylindole (DAPI)-stained cell nuclei were detected by Harmony software to find individual cells within all fields of view (Figure 4a). CellMask-stained cell cytoplasm defines the boundaries of all imaged cells. Only cells with whole cytoplasm within any imaged field of view were selected for subsequent analyses (green highlights in Figure 4b). Multiple markers were co-stained within the same cell (Figure 4c)—cell nuclei (blue, Figure 4c–f), cell cytoplasm (Figure 4c,d, red), filamentous actin (Figure 4e, green), and acetylated α-tubulin (Figure 4f, yellow). Post-translational tubulin acetylation is an indicator for increased microtubule stability, which can be quantified based on the amount of acetylated α-tubulin.^26^ Two-way ANOVA was used to estimate if cell morphology and cytoskeletal content were influenced by main effects of reelin exposure, disease status and if both main effects contributed to factorial interactions (reelin×disease status). Disease status significantly affected all measured parameters based on *post-hoc* Tukey’s test estimations (*P*<0.05): patient cells were smaller (Figure 4g), more round (Figure 4h), with less filamentous actin (Figure 4i), and less acetylated α-tubulin (Figure 4j). Reelin supplementation did not stimulate any changes to cell cytoskeleton and morphology (*P*>0.05).

### Patient cells were unable to modulate focal adhesions in response to extracellular reelin

**Figure 5:**
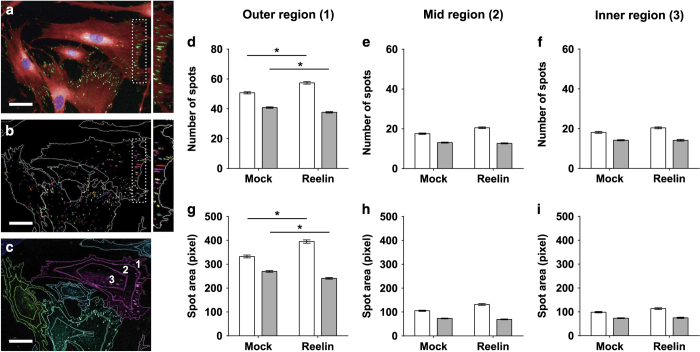
Patient-derived cells were unable to generate sufficient focal adhesions of the correct size on extracellular reelin. All cells were fixed and stained with primary antibody against phosphorylated FAK at tyrosine-397 (phospho-FAK Y397) followed by fluorophore-conjugated secondary antibody Alexa Fluor 488. Cell nuclei and cytoplasm were stained with DAPI and CellMask Deep Red, respectively. Images were captured on the Operetta High Content Imaging System and analyzed by Harmony software. Age-matched patient cell lines (*N*=9) were compared with control cell lines (*N*=9). For all individual cell lines, 300 single cells were quantified and grouped into subsequent disease or non-diseased dataset. (**a**) Representative immunocytochemical co-staining with multiple probes and markers for cell nuclei (blue), cell cytoplasm (red), and focal adhesions (green spots). Focal adhesion spots were elongated and larger at the cell periphery (insert). (**b**) Harmony software was used to locate and measure focal adhesion properties. DAPI-stained nuclei were used to locate individual cells within all imaged fields of view. “Find spot” algorithm was used to locate all focal adhesions within the boundaries of the cell determined by CellMask staining that stains for the cell cytoplasm. Harmony software identifies all focal adhesions as spots (insert). (**c**) Individual cells were divided into three cell regions based on relative distance away from the center of the cell defined by the nuclei stain. Harmony spot analysis generated two key parameter outputs—number of spots (number of focal adhesions; **d**–**f**) and spot size (focal adhesion size; **g**–**i**). All cells were divided into three regions—outer region (**d** and **g**), mid region (**e** and **h**) and inner region (**f** and **i**). All plotted data represents mean of all cells measured per group±s.e.m. Open bars represent control cell dataset; Closed gray bars represent patient cell dataset. *P<0.05 based on *post-hoc* Tukey’s test following two-way ANOVA. Scale bar = 50 μm.

Finally, we were interested to know if intracellular sensing capabilities were affected in patient cells. Phospho-FAK Y397 antibody was used to stain focal adhesions, which appear as spot-like structures (green spots, Figure 5a, insert on right) within cell cytoplasm (CellMask-stained, red, Figure 5a). Focal adhesions are predominantly localized at the outer peripheral region (insert, Figure 5a,b). Mature focal adhesions are FAK-containing elongated spots with an area of 1–10 μm^2^,^16^ which are large enough to be imaged on Operetta High Content Imaging System (PerkinElmer, Llantrisant, UK) at 20× magnification. At least 300 cells were quantified for each cell line to generate data in Figure 5d–i. We divided all imaged cells into three consistently defined regions presented in Figure 5c—(label 1) outer region, (label 2) mid region, and (label 3) inner region, and as expected, there were three times as many spots in the outer region (Figure 5d) compared with the mid (Figure 5e) and inner regions (Figure 5f). Functional focal adhesion activity occurs mostly in the peripheral ends of cells where cells “feel” and explore surrounding microenvironment via lamellipodia extensions.^16^ Therefore, only spots in the outer region were considered. Focal adhesion spots were detected by user-defined algorithms (Figure 5c) and quantified for two output parameters that relate to spot property: number of spots (Figure 5d–f) and relative spot size (Figure 5g–i).

Two-way ANOVA was used to test the main effects of disease condition and reelin on focal adhesion spot properties. Both disease status (F(1,10934)=595.2; *P*=4.73E−128) and exposure to reelin (F(1,10934)=8.3; *P*=0.004) contributed to significant deviations in the number of spots in patient cells compared with control cells. Similarly, spot size was affected by disease status (F(1,10934)=462.7; *P*=1.48E^−100^) and reelin (F(1,10934)=11.21; *P*=0.0008). There were significant factorial interactions between disease association and reelin stimulation to collectively cause aberrations to both spot numbers (F(1,10934)=65; *P*=8.48E−16) and spot sizes (*F*(1,10934)=82.3; *P*=1.36E^−19^). *Post-hoc* Tukey’s multiple comparisons test was used to estimate differences between measured groups (i.e., patient–control comparisons and with/without reelin coating). In mock-conditioned medium alone, patient cells had significantly fewer focal adhesions (mean=40.83±0.47 spots) compared with control cells (mean=50.76±0.72 spots) (*P*<0.05). Focal adhesions were also smaller in patient cells (269.90±3.65 relative size) compared with control cells (332.55±6.00 relative size) (*P*<0.05). Control cells responded to reelin by increasing the number (57.41±0.79 spots) and size (395.11±7.07 relative size) of focal adhesions (*P*<0.05). By contrast, reelin induced a small decrease in focal adhesion number (37.68±0.47 spots) and size (241.05±3.64 relative size; *P*<0.05) in patient cells.

### Patient cell motility was unaffected by antipsychotic drug treatments

We were interested to see if there was a correlation between antipsychotic medication used by individuals at the time of nasal biopsy with observations in this study (cell motility, focal adhesion size, focal adhesion number, cell morphology, F-actin, acetylated α-tubulin). Because all individuals were using different antipsychotic drugs of varying dosages, the medication dose at the time of biopsy was standardized by conversion to chlorpromazine equivalent doses (CED).^27^ Only *N*=8 cell lines were used for this analysis as risperidone dosage used by individual 30002009 was unknown during biopsy (Table 1). D’Agostino and Pearson omnibus normality test was used to check if data was normally distributed. Pearson correlation was used for normally distributed datasets to estimate linear relationship between measured variable and CED dosage. Otherwise, Spearman correlation was used for datasets that did not pass the normality test. There were no significant association between patient antipsychotic drug medication and any measurements in either mock or reelin-supplemented conditions in this study (*P*>0.05).

CyQUANT cell viability assay was used to find antipsychotic medication dosages that were not toxic to the cells. Two-way ANOVA was used to test the effects antipsychotic drug treatment and disease status on cell viability. Increasing antipsychotic drug caused reduced cell viability (*P*<0.05). However, disease status was not associated with reduced cell viability (*P*>0.05), with patient and control cell lines equally sensitive to increasing antipsychotic drug concentrations. *Post-hoc* Tukey’s multiple comparisons test estimated that cell viability significantly deviated from untreated DMSO at 100 μmol/l for clozapine and haloperidol (*P*<0.05; Supplementary Figure 1a,b), and 10 μmol/l for chlorpromazine treatment (*P*<0.05; Supplementary Figure 1c). To avoid excessive cell death, we used 20 μmol/l (clozapine and haloperidol) and 2 μmol/l (chlorpromazine) dosages for the subsequent cell motility assay.

Antipsychotic drug treatment reduced the motility of control cells but not patient cells (Supplementary Figure 1d). By using a two-way ANOVA, we found that disease status (F(1,3576)=39.94; *P*<0.0001) and antipsychotic drug treatment (F(3,3576)=10.29; *P*<0.00001) were significant factors that modulate cell motility. *Post-hoc* Tukey’s test indicated that track lengths of control cells deviated significantly from DMSO untreated conditions after all three antipsychotic drug treatments (*P*<0.05). In contrast, patient cell motility remained unchanged in the presence of antipsychotic drugs (*P*>0.05). Tukey’s test also identified significant disease differences in control cell motility after antipsychotic drug treatment (*P*<0.05 for all three antipsychotic drugs).

## Discussion

This study demonstrates that schizophrenia patient-derived cells were deficient in reelin-dependent cell motility and focal adhesion formation. Control cells reduced their motility when exposed to extracellular reelin, whereas patient cells did not alter their motility. Patient cells also expressed less endogenous reelin than control cells, just as reduced reelin mRNA and protein is reported in schizophrenia-derived cells^24^ and post-mortem brain tissue.^5–8^ Reduced reelin expression in post-mortem brain tissue led to the hypothesis that neuronal migration would be impaired in the developing brain in schizophrenia.^28^ This is the first study to show a link between reduced reelin and functional deficits in cell migration in schizophrenia.

To begin to understand the mechanism of the failure of patient cells to respond to reelin we looked at the early components of the reelin signaling pathway. The motility deficit was not due to a lack of the reelin signaling pathway because patient and control cells expressed similar levels of the reelin receptors, ApoER2 and VLDLR, and similar levels of the Dab1 adaptor protein, which is phosphorylated by binding of reelin with its receptors. Cell movement requires cells to attach to the ECM via membrane protein complexes called focal adhesions. We showed previously that these patient cells have fewer and smaller focal adhesions than control cells, with faster dynamics of focal adhesions.^17^ In this study we show that extracellular reelin stimulated control cells, but not patient cells, to increase the number and size of their focal adhesions, indicating that in patient cells, reelin signaling was not transduced into the final cellular effector of motility, focal adhesions.

Focal adhesions are essential in cell migration,^29^ being the membrane components at the edges of the cell that link intracellular actin filaments to ECM proteins via integrin receptors.^16^ Interestingly, reelin can affect this mechanism directly because reelin binds integrin α3β1 in a complex with the other reelin receptors—ApoER2 and VLDLR, to inhibit cell migration.^14^ In addition, the reelin signaling protein Dab1 aggregates at the cytoplasmic tail of integrin β1.^30^ Reelin signaling has also been shown to “crosstalk” with integrin signaling, to activate integrin α5β1 binding to fibronectin within the brain.^15^ Taken together, these observations indicate that reelin may act directly on the integrin-focal adhesion signaling cascade as well as as via unknown mechanisms via the traditional reelin signaling cascade. In support of this is our unpublished evidence that patient and control cells express similar levels of α3, α5, and β1 integrin receptors, as well as a whole panel of common integrin receptors, to suggest that reelin binding could potentially modulate cell motility in these cells via integrin and FAK signaling. Focal adhesions were dysregulated in two ways in patient cells. Patient cells had fewer and smaller focal adhesions that did not increase upon reelin exposure compared with control cells. In fact, there was a small reelin-induced decrease in the size and number of focal adhesions in patient cells. The reelin-induced increase in focal adhesions in control cells was thus associated with a decrease in motility, whereas there was no change in motility in patient cells and a small decrease in focal adhesions. Although the baseline difference between patient and control cells might suggest that more focal adhesions equates to more motility,^31^ in control cells reelin increased focal adhesion number and size but decreased motility. This highlights the complexity of cellular capabilities to modulate the mechanics of cell migration.^32^ For example, there is a “Gaussian biphasic relationship” between focal adhesion size and cell motility speed, where further increments in focal adhesion size will no longer stimulate cells to move faster after a certain maximal threshold, but instead reduces cell motility.^33^ The core cellular deficits in patient cells may thus be in regulation of the dynamics of regulatory pathways affecting cell motility such as the differences in the dynamics of focal adhesion turnover previously observed in these cells.^17^ The FAK pathway can be regulated at many levels. Our findings indicate possible global defects in cell motility, which provides a platform for testing the roles of specific proteins, such as paxillin or talin, components of focal adhesions, in reelin-associated deficits in schizophrenia.

Published studies show that reelin signaling modulates neuronal cytoskeletal dynamics via regulator proteins that directly interact with actin and microtubules. Reelin binding to its receptors phosphorylates and activates Dab1. This then activates n-cofilin^20^ and microtubule-associated protein 1B (MAP1B),^34^ which stabilizes the actin cytoskeleton and promotes actin-microtubule crosstalk, respectively. Reelin binding also activates the Rho GTPase, Cdc42, which promotes actin polymerization to increase filopodia at cell leading edge^23^ and inhibits lamellipodium formation.^20^ In the present study patient cells had reduced baseline levels of both of these essential components for cell motility: less F-actin, a measure of polymerized actin, and less acetylated α-tubulin, a measure of stabilized microtubules. Extracellular reelin did not affect the expression of these cytoskeleton components in either patient or control cells. This means defective focal adhesion could be the key driver for non-responsive patient cell migration phenotype. However, we cannot rule out a possibility that cytoskeletal dynamics may be affected in patient cells.

Schizophrenia patient brains show widespread irregularities in total size and regional volume.^35,36^ These differences are strong neuropathological evidence for dysregulated neuronal migration and positioning during development,^37^ which are important for correct formation of the intricate compartments of the human brain. Our functional evidence for deficits in reelin-induced cell motility and reduced levels of reelin in patient cells strengthens the hypothesis that reelin deficiency in the schizophrenia brain is indicative that deficiencies in reelin signaling may contribute to cytoarchitectural irregularities affecting neurobiological processes controlling cognition and neurotransmission. In the context of schizophrenia, the reeler mouse mutant brain offers indicative links with neuropathological analyses of patient brains which have reduced levels of reelin in the cortex and hippocampus.^28,38^ For example, reeler brains have peculiar structural defects in the cortices and hippocampus^39^ and schizophrenia patient brains have a thinner cerebral cortex^40^ and reduced hippocampal volume.^36^ Besides its role in neuronal migration, reelin also stimulates formation of dendrites^41^ and synapses^42^ during neurodevelopment. Moreover, reelin continues to regulate synaptic remodeling in the adult brain.^43^ Reelin supplementation into brains of wild-type mice increased dendritic density and synapse potentiation, to improve “cognitive ability”.^44^ It is important to realize that the difference in cortical lamination in the reeler mutant brain is far more severe than in the schizophrenia post-mortem brain. Moreover, unlike in reeler, reelin protein was still detected in schizophrenia patient brains, albeit at a lower level compared with healthy controls. In accord with evidence that many genes are implicated in schizophrenia,^45^ cytoarchitectural changes in the schizophrenia brain would be expected to be milder than in reeler mice, probably resulting from small alterations to multiple signaling pathways, compared with large effects from mutations in the *RELN* gene as reported in autosomal recessive lissencephaly.^46^

### Technical considerations

Patient-derived olfactory cells are considered here as representative disease models to study cellular mechanisms of reelin function in cell migration. Being sourced from live patients, this cell model encompasses the polygenic nature of schizophrenia, in contrast to simpler *RELN*-knockout cell and animal models. The greater complexity and variability involved in comparing patient and control cells was obviated through the use of fully automated imaging and analysis in multi-well format assays allowing unbiased quantification across large numbers of cells from multiple patients and controls under different experimental conditions.

Like other patient-derived models, the present study may be confounded by exposures to smoking and antipsychotic medications at the time of biopsy. These confounds are common to cell-based and post-mortem studies, which are necessarily limited in sample size and difficult to match for other than age and sex. On the other hand, there was no influence of these confounds in gene or protein expression in these cells^24,47^ or in other assays of cell function.^17,24^ In this study, there was no correlation between antipsychotic medication dose at the time of nasal biopsy and any cell measurement (cell motility, focal adhesion size, focal adhesion number, cell morphology, F-actin, acetylated α-tubulin). It is possible that prior antipsychotic treatment of patients could affect the responses of their cells to antipsychotics presented *in vitro* for example via DNA methylation.^48^ We found that patient cell motility was not affected by any of the three antipsychotic medications (clozapine, haloperidol, chlorpromazine). In contrast, all medications reduced the motility of control cells. We cannot rule out that previous medication history in the patients subsequently affected the motility of their cells. On the other hand, the lack of patient cell response may reflect a global deficit in regulation of motility in response to extracellular signals such as reelin and other ECM proteins (unpublished data).

Although olfactory neurosphere-derived stem cells are neural in origin,^49^ they are not differentiated neurons or glia and hence may not completely mimic neuronal subpopulations in the schizophrenia brain. Nonetheless, the present results are consistent with current hypotheses based on post-mortem brain and known functions of reelin, to provide concrete hypotheses for further testing in other patient-derived cell models such as neurons and neural precursors generated from induced pluripotent stem cells.^50–52^ The consequences of reelin signaling deficits on the structure of the developing human brain may require more complex patient-derived *in vitro* organoid models,^53^ which comprise neural cells differentiated from induced pluripotent stem cells derived from human fibroblasts. These “mini-brains” can be grown in a three-dimensional culture system and form various brain regions, such as neocortex,^53^ which could potentially be used to verify *in vitro* the role of reelin in early events of human brain development.

## Concluding remarks

Our results demonstrate for the first time, reelin-dependent functional deficits in cells derived from patients with schizophrenia. Patient cells had less reelin and unlike control cells, were not responsive to extracellular reelin. Although they were not reelin-dependent, several aspects of patient cell biology were reduced compared with control cells. Patient cells were smaller; they expressed less filamentous actin and less acetylated α-tubulin, a marker of stable microtubules; and they had fewer and smaller focal adhesions. These differences point to baseline differences that could affect the regulatory landscape of cell motility. For example, if the availability of cytoskeletal proteins were reduced, the patient cell’s ability to regulate the dynamics changes necessary for changing cell adhesion and motility could be limited. Similarly, baseline differences in focal adhesion dynamics^17^ may also affect the ability of patient cells to respond appropriately to the extracellular environment, which is the case for the ECM glycoprotein in reelin based on our present findings.

## Materials and methods

### Olfactory neurosphere-derived cells

All cell lines used were from the same cohort as previous studies.^17,24,25^ Control line (ID: C1 100030002) has been replaced with C1 10008017. Cell lines were expanded from olfactory mucosa biopsies from age-matched male donors, which included nine schizophrenia patients and nine healthy controls (Table 1). The patient group was classified based on the Diagnostic Interview for Psychosis (DIP), according to the Diagnostic and Statistical Manual of Mental Disorders IV (DSM-IV). Human biopsies were collected in compliance with The Park Centre for Mental Health and Griffith University ethics committees. All participants gave written, informed consent for their cells to be grown *in vitro*, banked and used for experiments to understand the biological bases of schizophrenia. All experiments were compliant with guidelines from the National Health and Medical Research Council of Australia.

### Cell culture and synchronization

Cell lines from patient biopsies and healthy control biopsies were referred to as “patient cells” and “control cells”, respectively. All cells were maintained and passaged as previously described.^24^ Cells between passages 5–10 were grown in Dulbecco’s Modified Minimum Essential Medium (DMEM/F12; Gibco, Life Technologies, Carlsbad, CA, USA) supplemented with 1% penicillin–streptomycin (Gibco, Life Technologies) and 10% fetal bovine serum (Bovogen, Keilor East, VIC, Australia). Cells used in all cytological screening assays were synchronized by serum deprivation before re-entering the cell cycle at G1 phase.^17^

### Western blot

Total protein lysates were extracted from cells using lysis buffer containing: 50 mmol/l Tris–HCl, 1 mmol/l EDTA, 150 mmol/l NaCl, 1% Triton X-100, 5 mmol/l NaF, 0.25% sodium deoxycholate, 2 mmol/l Na_3_VO_4_ and 1× Roche Complete Protease Inhibitor Cocktail (Roche, Basel, Switzerland). Cell lysates were passed through a 27 G needle followed by centrifugation at 21,130*g* for 15 min at 4 °C. Total protein samples were fractionated using Tris-glycine SDS–PAGE in a 5% stacking polyacrylamide gel followed by a 6% (for reelin) or 12% (for all other proteins) resolving polyacrylamide gel at 100 V for 2 h. Fractionated proteins were transferred onto nitocellulose membrane in 2× Tris-glycine transfer buffer with 10% (v/v) methanol at 25 V for 20 h, followed by 70 V for 3 h at 4 °C (*reelin*) or 1× Tris-glycine transfer buffer with 20% methanol at 100 V for 3 h (all other proteins). Membranes were blocked in 5% skim milk for 1 h, followed by incubation with primary antibodies: mouse anti-Reelin antibody (1:500, a.a.164–189 mreelin, clone 142; Merck Millipore, Billerica, MA, USA), anti-Dab1 (1:1,000; Abcam, Cambridge, UK), anti-ApoER2 (1:200; Abcam), anti-VLDLR (1:200, clone 6A6, Santa Cruz Biotechnology, Dallas, TX, USA). Anti-β-tubulin antibody (1:5,000; Sigma Aldrich, St Louis, MO, USA) was used as a loading control for all markers. Membranes were washed and incubated in HRP-conjugated anti-mouse or HRP-conjugated anti-rabbit secondary antibody (both at 1:10,000; Sigma Aldrich) for 45 min. Membranes were washed and incubated with Immobilon Western Chemiluminescent HRP substrate (Merck Millipore) for 5 min. Chemiluminescence signal was detected and analyzed using Quantity One 1-D software (BioRad, Hercules, CA, USA).

### Flow cytometry

Cells cultured to approximately 90% confluence in a 75 cm^2^ flask were trypsinized with TrypLE (Gibco, Life Technologies). Single cell suspensions were fixed with 4% paraformaldehyde (PFA; in Hank’s Balanced Salt Solution (HBSS); Gibco, Life Technologies) for 15 min. Cells were washed in flow buffer, made up of 1% BSA and 0.1% Triton X-100 in Dulbecco’s phosphate-buffered saline (DBPS; Gibco, Life Technologies) and centrifuged at 0.3*g* for 5 min. Fixed cells were permeabilized and blocked with 1% BSA and 0.1% Triton X-100 for 30 min. Cells were stained with anti-reelin antibody for 1 h (1 μg antibody/1×10^6^ cells; a.a.164–496 mreelin, clone G10; Merck Millipore), followed by rabbit anti-mouse Alexa Fluor 488 for 30 min (Life Technologies). Concentration-matched mouse IgG1 was used as the isotype negative control (Merck Millipore). Fluorescence staining was measured by recording 10,000 events via FACSAria cytometer (BD; Franklin Lakes, NJ, USA). Reelin staining was quantified relative to isotype control measurements using Summit Version 4.3.02 (Beckman Coulter, Brea, CA, USA).

### Full-length reelin protein production

HEK293FT cells were transfected with pCrl, plasmid containing full-length reelin sequence,^12^ by electroporation with Amaxa Nucleofector Kit V (Lonza, Basel, Switzerland) according to the manufacturer’s protocol, and allowed to adhere on 75 cm^2^ flasks pre-coated with poly-l-lysine (Sigma Aldrich, Saint Louis, MO, USA). After 4 h, growth medium was replaced with serum-free basal medium containing DMEM/F12 and penicillin–streptomycin. Conditioned medium was collected and snap frozen at −80 °C every 24 h for 6 days. Conditioned medium were combined at the end and filter purified with Amicon-15 centrifugal units according to the manufacturer’s instructions (Merck Millipore). The exact protocol was conducted concurrently with mock vector pcDNA3.1 to produce mock-conditioned medium, which was used as assay control for all experiments. Western blot was used to detect and validate full-length reelin protein. Full-length reelin concentrations were estimated by using a laminin standard curve with known laminin concentrations (mouse laminin; Gibco, Life Technologies). Purified reelin conditioned medium was diluted in UltraPure Water (Gibco, Life Technologies) to give a final reelin concentration of 5 μg/ml; 40 μl reelin protein solution was charged into selected wells of a 96-well CellCarrier plate (Perkin Elmer, Waltham, MA, USA) and incubated for 16 h at room temperature to coat plastic surfaces. All wells were washed and air dried for 2 h before cell seeding.

### Single cell motility assay

Both patient and control cells were seeded into corresponding reelin or mock wells at 2,500 cells/well and allowed to attach onto well surfaces. Live cell nuclei were stained with NucBlue Live ReadyProbes reagent (Molecular Probes, Life Technologies). All wells were imaged with 360–400 nm excitation wavelength to visualize the nuclei dye using Operetta High Content Imaging System (Perkin Elmer, Llantrisant, UK). Operetta was programmed to maintain the live cell chamber at optimal cell growth conditions (37 °C; 5% CO_2_) and to capture an image for 11 fields of view per cell line and coating type at 30 min intervals for total duration of 24 h. Time-lapse image sequences were computed by tracking *x*–*y* coordinates via Operetta, followed by batch analysis of resulting accumulated track length using the Harmony High Content Imaging and Analysis Software (Perkin Elmer). Cell nuclei were used as a reference point for each individual cell and tracked over 24 h to compute track data. Only cells that have been continuously tracked for the whole duration were considered for final analyses.

### Fixed-cell cytological imaging and analysis

Final cell densities of 2,500 cells per well were seeded into 96-well CellCarrier plates pre-coated with either reelin or mock-conditioned medium. All cells were incubated for 24 h and fixed with 4% PFA for 15 min at room temperature. Fixed cells were probed for filamentous actin (Alexa Fluor 488 Phalloidin; 1:100; Life Technologies), acetylated α-tubulin (1:200; Santa Cruz, clone 6-11B-1), and phospho-FAK Tyr397 (1:100; Cell Signaling Technology, Danvers, MA, USA). Fluorophore-conjugated secondary antibodies used were: Alexa Fluor 546 (1:400; Life Technologies; for acetylated α-tubulin) and Alexa Fluor 488 (1:400; Life Technologies; for phospho-FAK Tyr397). Markers for actin and acetylated α-tubulin were co-stained in the same cell, whereas phospho-FAK Tyr397 was stained separately. Nuclei and cell cytoplasm were stained with DAPI (1:1,000; Life Technologies) and CellMask Deep Red Plasma Membrane Stain (1:5,000; Life Technologies), respectively. Multiple fields of views within all wells were imaged at 20× objective magnification on the Operetta at four different excitation wavelengths depending on fluorophores used. Resulting images were analyzed by Harmony software programmed with user-defined analytical workflows to measure parameters such as cell morphology (area and roundness), fluorescence staining intensities (actin and acetylated α-tubulin) and spot properties (for phospho-FAK).

### Antipsychotic medications and cell motility

Patient and control cells were seeded into wells of 96-well plate and treated with 1, 10, and 100 μmol/l of clozapine (C6503), haloperidol (H1512), and chlorpromazine hydrochloride (C1813) for 24 h. All drugs were from Sigma Aldrich and reconstituted in dimethyl sulfoxide (DMSO, Sigma Aldrich). Initial toxicity assays were undertaken to select appropriate concentrations to use for subsequent cell motility assays. Cell viability was measured using CyQUANT NF Cell Proliferation Assay (Life Technologies) according to the manufacturer’s instructions. Fluorescence measurements were quantified on BioTek plate reader and were standardized to assay blanks containing DMSO only, and then normalized to the untreated control. Cell motility was quantified using the same parameters and protocol described above. Antipsychotic medications were supplemented into growth medium during the whole duration of the tracking assay at the following concentrations: 20 μmol/l clozapine, 20 μmol/l haloperidol and 2 μmol/l chlorpromazine hydrochloride.

### Statistical analysis

All statistical analyses were conducted using GraphPad Prism (version 6.05, GraphPad Software, Inc., La Jolla, CA, USA). Raw data were grouped into a dataset comprised of patient cell and control cell measurements, to obtain mean and standard errors (s.e.m.). Non-parametric, two-tailed student’s *t*-test was used to estimate simple patient–control differences. Two-way analysis of variance (ANOVA) test was conducted on datasets with two variables (i.e., disease status and reelin supplementation), to estimate main effects and interaction of variables on dependent variables (expression levels, size, roundness, spot number and spot size). *Post-hoc* multiple comparisons Tukey’s test was performed to identify significant deviations between two groups (patient–control or with/without reelin). Testing is considered significant if the *P* value is less than or equal to the alpha (*P*<0.05).

## Figures and Tables

**Table 1:** Participant details

*Cell line ID*	*Age*	*Sex*	*Medication*	*CED*	*Cigarettes/day*
*CONTROLS*					
10008017	49	Male			
10002001	31	Male			
10002002	47	Male			10
10002003	28	Male			
10003001	17	Male			
10003003	32	Male			
10003004	46	Male			
10003005	56	Male			
10003006	45	Male			5
					
*PATIENTS*					
30002001	46	Male	Clozapine: 250 mg/day	333	25
			Omeprazole magnesium: 20 mg/day		
30002002	58	Male	Olanzapine: 7.5 mg/day	250	
			Benztropine: 1 mg/day		
			Diclofenac sodium: 100 mg/day		
30002003	21	Male	Quetiapine: 800 mg/day	1,194	15
			Paroxetine: 40 mg/day		
30002004	33	Male	Risperidone: 4 mg/day	267	
30002005	49	Male	Clozapine: 350 mg/day	467	
30002006	27	Male	Olanzapine: 16 mg/day	533	30
30002007	44	Male	Clozapine: 475 mg/day	633	20
			Lithium carbonate: 1,250 mg/day		
			Atenolol: 75 mg/day		
			Aspirin: dose unknown		
30002008	28	Male	Flupenthixol decanoate: 200 mg/month	500	10
30002009	38	Male	Risperidone: dose unknown	Unknown	60

Abbreviation: CED, chlorpromazine equivalent dose.

Table adapted from Fan *et al.*;^17^ data originally published in Matigian *et al.*^24^
