# Supplementary figures and images for: Schizophrenia patient-derived olfactory neurosphere-derived cells do not respond to extracellular reelin
Source: NPJ Schizophr. 2016 Aug 17;2:16027–. doi: 10.1038/npjschz.2016.27 (PMC4994154; doi:10.1038/npjschz.2016.27)

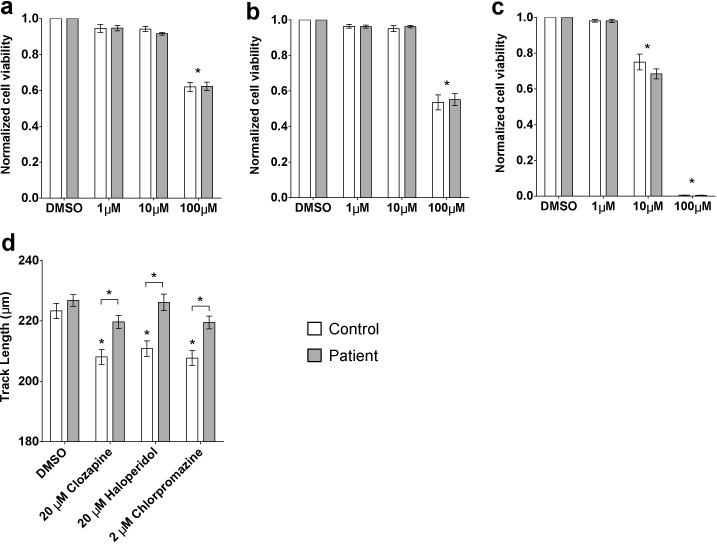

Supplement: Supplementary Figure 1 [file npjschz201627-s1.jpg]
